# Aliskiren and Amlodipine in the Management of Essential Hypertension: Meta-Analysis of Randomized Controlled Trials

**DOI:** 10.1371/journal.pone.0070111

**Published:** 2013-07-29

**Authors:** Yukai Liu, Ken Chen, Xun Kou, Yu Han, Lin Zhou, Chunyu Zeng

**Affiliations:** 1 Department of Cardiology, Daping Hospital, The Third Military Medical University, Chongqing, P. R. China; 2 Chongqing Institute of Cardiology, Chongqing, P. R. China; Royal College of Surgeons, Ireland

## Abstract

**Background:**

Aliskiren is a novel renin-angiotensin aldosterone system (RAAS) inhibitor, the combination therapy of aliskiren and amlodipine for blood pressure control have been reported recently. The primary objective of this analysis is to review recently reported randomized controlled trials (RCTs) to compare antihypertensive effects and adverse events between mono (amlodipine or aliskiren alone) and combination therapy of both medicines.

**Methods:**

Databases for the search included Pubmed, Embase and the Cochrane Central Register of Controlled Trials. Revman v5.0 statistical program was used to analyze the data. Weighted mean differences (WMD) with a 95% confidence interval (CI) were used for the calculation of continuous data, and relative risk (RR) with a 95% CI was used for dichotomous data.

**Results:**

We analyzed the data from 7 RCTs for a total of 6074 participants in this meta-analysis. We found that the aliskiren/amlodipine combination therapy had a stronger effect in lowering blood pressure as compared with the monotherapy using aliskiren (SBP: WMD = −10.42, 95% CI −13.03∼−7.82, P<0.00001; DBP: WMD = −6.60, 95% CI −7.22∼−5.97, P<0.00001) or amlodipine (SBP: WMD = −4.85, 95% CI −6.88∼−2.81, P<0.00001; DBP: WMD = −2.91, 95% CI −3.85∼−1.97, P<0.00001). No differences were found in terms of adverse events between combination therapy and monotherapy, except for the rates of peripheral edema and hypokalaemia which were significantly lower in the combination therapy than in the amlodipine monotherapy (RR = 0.78, 0.66∼0.92, P = 0.004; RR = 0.51, 0.27∼0.97, P = 0.04). Similar antihypertensive effects were found in both obese (body mass index > = 30 kg/m^2^) hypertensive and non-obese (body mass index <30 kg/m^2^) hypertensive patients. Moreover, there was no difference with the blood pressure lowering or adverse effects with regards to the combination therapy in both subgroups.

**Conclusion:**

We found that aliskiren/amlodipine combination therapy provided a more effective blood pressure reduction than monotherapy with either drug without increase in the occurrence of adverse events.

## Introduction

Hypertension is a highly prevalent world-wide health problem, and is a major risk factor of cardiovascular disease. There is strong evidence showing that the increase in blood pressure is associated with stroke, heart and renal failure. Reducing the elevated blood pressure could improve cardiovascular outcome [1].

Calcium channel blockers (CCB) and renin–angiotensin aldosterone system (RAAS) inhibitors are effective medicines for the treatment of hypertension. Amlodipine, one of the CCBs, is a widely used medicine for hypertension, by inhibiting calcium ions influx through the L-type calcium channels of vascular smooth muscles, and thereby directly causes vasodilation. In addition, amlodipine also provides cardiovascular disease prevention [2], [3] and is commonly used alone or in combination with other antihypertensive medicines. RAAS inhibitors are another effective class of blood pressure medicines that plays a key role in blood pressure regulation and water-electrolyte metabolism. Excessive activity of RAAS may increase blood pressure (BP) and exert direct growth-promoting effects on tissues, which can lead to end-organ damage [4], [5]. Therefore, blockade of RAAS could reduce blood pressure and protect the target organs, including the heart, kidney and brain.

Aliskiren is a direct renin inhibitor (DRI) that blocks the RAAS at its first rate-limiting step, by blocking the conversion of angiotensinogen to angiotensin I, thus inhibiting plasma renin activity (PRA) and reducing the production of angiotensin II and aldosterone [6]. As the first of a new class of orally-taken renin inhibitors, aliskiren was approved for the treatment of hypertension by the U.S. Food and Drug Administration in 2007, and proved to be effective in blood pressure control [7]. Researchers further found that aliskiren could provide more anti-hypertension efficacy when combined with other kinds of blood pressure medicines [8]–[10]. An increasing number of clinical trials have assessed the anti-hypertension efficacy and tolerability of aliskiren, amlodipine, and combination therapy of both medicines. However, due to the varying differences in patient number and other limitations, the conclusions drawn are not consistent, or even controversial. In this meta-analysis, we reviewed recently reported RCTs, and compared the antihypertensive effects and adverse events of monotherapy (amlodipine, or aliskiren) with those of combination of both medicines in general hypertensive patients and additional subgroups with obese (body mass index > = 30 kg/m^2^) and non-obese hypertensive patients.

## Methods

We followed the procedures described in the Cochrane Handbook for Systematic Reviews of Interventions and the Preferred Reporting Items for Systematic Reviews and Meta-Analyses (PRISMA) statement.

### Criteria of Trial Inclusion and Exclusion

The inclusion criteria of this review includes the requirements as follows: all the trials should be randomized controlled trials; the participants were all adults (age > = 18 years) with clear diagnosis of essential hypertension (SBP>140 mmHg, DBP>90 mmHg), and received aliskiren, amlodipine or combination treatment for hypertension; SBP and DBP reduction, adverse events are clearly measured in the articles, all the articles has clear description of withdrawals and dropouts. The exclusion criteria included the following requirements: the participants were diagnosed as secondary hypertension; trials were duplicated publications on the same group of patients; the study was an experimental trial; data in the article is missing or lacking.

### Search Strategy

We searched for the articles in the databases; PubMed (articles published from January 1980 to April 2013), EMBASE (articles published from January 1980 to April 2013) and the Cochrane Central Register of Controlled Trials (Cochrane Library Issue, 2012), for all relevant articles published in any language. The following medical subject heading terms and text words were used: aliskiren, renin inhibitor, amlodipine, calcium channel blocker blood pressure, adverse events. We limited our searches to randomized controlled trials in adults.

### Data Collection

Two investigators (Yukai Liu & Ken Chen) independently reviewed the literature searches to identify relevant trials that met the inclusion criteria, and any disagreement was resolved by extensive discussions. The following general descriptive information was extracted from each trial: authors; year of publication; trial design and interventions; the number and age of participants; baseline SBP and DBP values, and change from baseline of SBP and DBP; number of drop outs or withdrawals for any reason. The number of adverse events was also extracted.

### Quality Assessment

The quality of the included RCTs were assessed by Yukai Liu and Ken Chen using the Cochrane Collaboration’s Tool for assessing risk of bias [11]. Bias was evaluated in the following domain: adequate sequence generation; allocation concealment; blinding of research personnel; blinding of outcome; incomplete outcome data addressed; free of selective reporting; free of other bias. Each domain was graded as low, moderate, high risk of bias. Disagreements between the reviewers were resolved by discussion.

### Data Synthesis and Analysis

We analyzed 3 randomized controlled trials to compare the efficacy of 300/10 mg/d aliskiren/amlodipine combination therapy versus 300 mg/d aliskiren monotherapy, and 6 randomized controlled trials for the SBP and DBP reduction efficacy of aliskiren at dosage of 150 mg/d and 300 mg/d combined with amlodipine versus the amlodipine monotherapy. To determine whether there was a differential effect by aliskiren and amlodipine in obese- and non-obese hypertensive patients, we included two randomized controlled trials in obese patients (which was defined as body mass index > = 30 kg/m^2^) and non-obese patients (which was defined as body mass index <30 kg/m^2^), and compared the BP reduction efficiency of the combination therapy in obese patients with that in non-obese patients. We next compared safety of aliskiren combined with amlodipine versus alsikrein or amlodipine monotherapy. The incidence of adverse events (AEs) included peripheral edema, nasopharyngitis, dizziness, headache, diarrhea, hyperkalaemia (serum potassium>5.5) and hypokalaemia (serum potassium<3.5) [12]–[18]. Serious adverse events (SAEs) were defined as any event that was fatal or life-threatening, resulted in persistent or significant disability, constituted a congenital abnormality, required in-patient hospitalization or prolonged hospitalization, or was considered in some other way to be medically significant including myocardial infarction, cerebrovascular accident, gastroenteritis, pneumonia and retinal detachment [12]–[18]. Discontinuation due to the occurrence of adverse events related to the medications was also evaluated. All these AEs were reported in the overall population.

We used weighted mean differenced (WMD) and 95% confidence intervals (CIs) for continuous outcomes (SBP and DBP of participants in the trials) and relative risk (RR) which was obtained by χ^2^–test with 95% CIs for dichotomous outcomes (adverse events of participants in the trials). We tested the heterogeneity by using the χ^2^–test and I^2^ statistic across included trials. We considered I^2^<50% to indicate statistically significant heterogeneity. A fixed-effect model was elected when I^2^<50%, while a random-effect model was elected when I^2^>50%. Publication bias was evaluated by the funnel plot. The statistical computation for this meta-analysis was performed using Revman v5.0 (Revman, The Cochrane Collaboration, Oxford, UK).

## Results

### 1. Search Results

**Figure 1:**
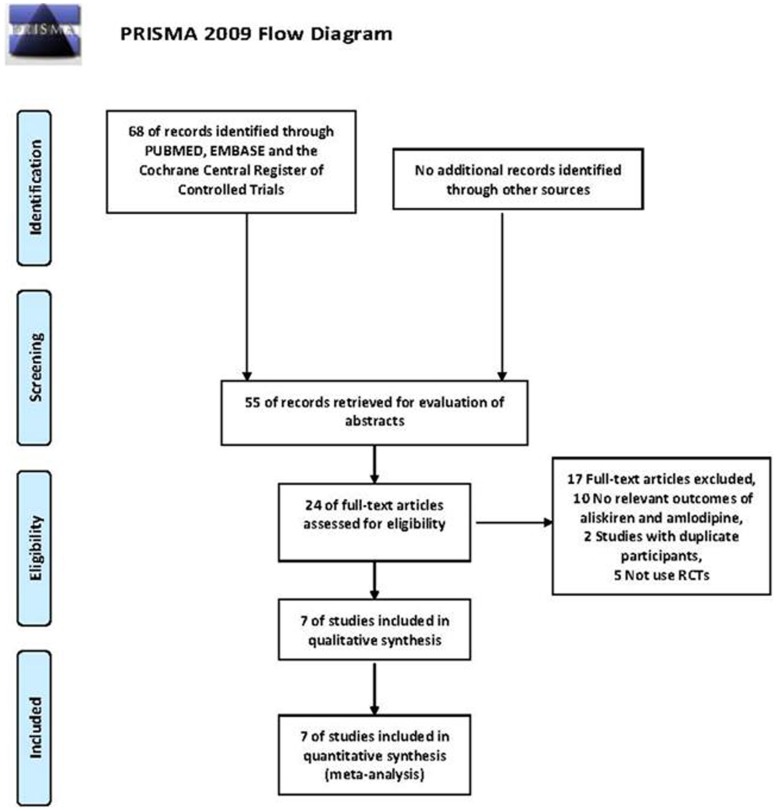
PRISMA flow diagram.

A total of 308 articles were identified via search of the databases Medline, Pubmed and Cochrane Central Register of Controlled Trials. 32 relevant articles were in-depth reviewed, and full texts were retrieved. In these articles, eligible studies included 7 randomized controlled trials, involving a total of 6074 participants [13]–[19], which met all the predetermined criteria. The quality of the included studies was with low risk and moderate risk of bias; the results were shown in **Table 1** and **Table S1**. **Figure 1** showed the results of the article search and selection flow chart, and **Table 1** shows the characteristics of the included studies.

**Table 1:** Characteristics of studies included in the meta-analysis.

Author	Publication date	Methods	Participants	Interventions	Assessment ofoutcome	Duration	Drop out	Risk of bias
Brown MJ[17]	2011	Multicenter double-blind, randomised, parallel-group, superiority trial	1247 participants, 315 patients were randomlyassigned to amlodipine 10 mg, 315 patientswere randomly assigned to aliskiren300 mg, and 617 to aliskiren/amlodipine300/10 mg, aged>18 years.	Combination therapy:aliskiren/amlodipine 150 mg/5 mg, 300 mg/10 mg, once daily. Monotherapy: amlodipine 5 mg, or 10 mg, oncedaily. Aliskiren 150 mg, or 300 mg,once daily.	BP reduction, rate of adverse events in total participants, laboratory evaluations.	8 weeks	188	Low risk
Weinberger MH[14]	2011	Prospective, multicenter, randomized, double-blind, parallel-group	443 participants, 223 patients were randomlyassigned to amlodipine, and 220 to aliskirenplus amlodipine, aged>18 years.	Combination therapy: aliskiren/amlodipine 300 mg/10 mg, oncedaily. Monotherapy: amlodipine10 mg, once daily.	BP reduction, rate of adverse events in total participants.	8 weeks	33	Moderate risk
Pfeiffer D[18]	2012	Multicenter, randomized, double-blind, parallel-group	847 participants, 283 patients were randomlyassigned to amlodipine, and 279 to aliskiren/amlodipine 300/10 mg, 285 to aliskiren/amlodipine 150/10 mg, aged>18 years.	Combination therapy: aliskiren/amlodipine 300 mg/10 mg,150/10 mg once daily. Monotherapy:amlodipine 10 mg once daily.	BP reduction, rate of adverse events in total participants, laboratory evaluations.	8 weeks	61	Low risk
Drummond W[13]	2007	Multicenter, randomized, double-blind, active-controlled, parallel-group study	545 participants, 180 patients were randomlyassigned to amlodipine 5 mg, 178 patientswere randomly assigned to amlodipine10 mg, and 187 to aliskiren plus amlodipine,aged>18 years.	Combination therapy:aliskiren/amlodipine 150 mg/5 mg, oncedaily. Monotherapy: amlodipine5 mg, or 10 mg, once daily.	BP reduction, rate of adverse events in total participants.	6 weeks	22	Moderate risk
Glorioso N[16]	2012	Multicenter, randomized, double-blind, parallel-group multicenter study	820 participants, 260 patients were randomlyassigned to aliskiren 300 mg, 277 patients wererandomly assigned to aliskiren/amlodipine300/5 mg, and 283 to aliskiren/amlodipine300/10 mg, aged>18 years.	Combination therapy:aliskiren/amlodipine 300 mg/5 mg,300/10 mg, once daily. Monotherapy:aliskiren 10 mg, once daily.	BP reduction, rate of adverse events in total participants, laboratory evaluations.	8 weeks	41	Low risk
Braun-DullaeusRC [15]	2012	Multicentre, randomized, double-blind study	485 participants, 241 patients were randomlyassigned to amlodipine 10 mg, and 244 toaliskiren/amlodipine 300/10 mg,aged>18 years.	Combination therapy: aliskiren/amlodipine 300 mg/10 mg, oncedaily. Monotherapy: amlodipine10 mg, once daily.	BP reduction, rate of adverse events in total participants, laboratory evaluations.	7 weeks	52	Moderate risk
LittlejohnTW[19]	2012	Multicentre, randomized, double-blind, placebo-controlled	1687 participants, 198 participants wererandomly assigned to placebo; 195 participants wererandomly assigned to aliskiren 150 mg, 203participants to aliskiren 300 mg; 185 participantswere randomly assigned to amlodipine 5 mgand 181 to amlodipine 10 mg; 181 participantswere randomly assigned to aliskiren/amlodipine150/5 mg, 183 participants to aliskiren/amlodipine150/10 mg, 178 participants to aliskiren/amlodipine300/5 mg, and 183 participants to aliskiren/amlodipine300/10 mg, aged>18 years.	Combination therapy: aliskiren/amlodipine 300/5 mg, 300/10 mg,150/5 mg, 150/10 mg once daily.Monotherapy: amlodipine 5 mg,10 mg; aliskiren 150 mg,300 mg once daily.	BP reduction, rate of adverse events in total participants, laboratory evaluations.	8 weeks	149	Low risk

### 2. Publication Bias Analysis

A funnel plot for the studies in each group was shown in **Figure S1.** No significant asymmetric differences were found in any of the funnel plot results, indicating no significant publication bias among the studies.

### 3. Effect of Lowering Blood Pressure

#### 3.1. Combination therapy of aliskiren combined with amlodipine versus aliskiren

**Figure 2:**
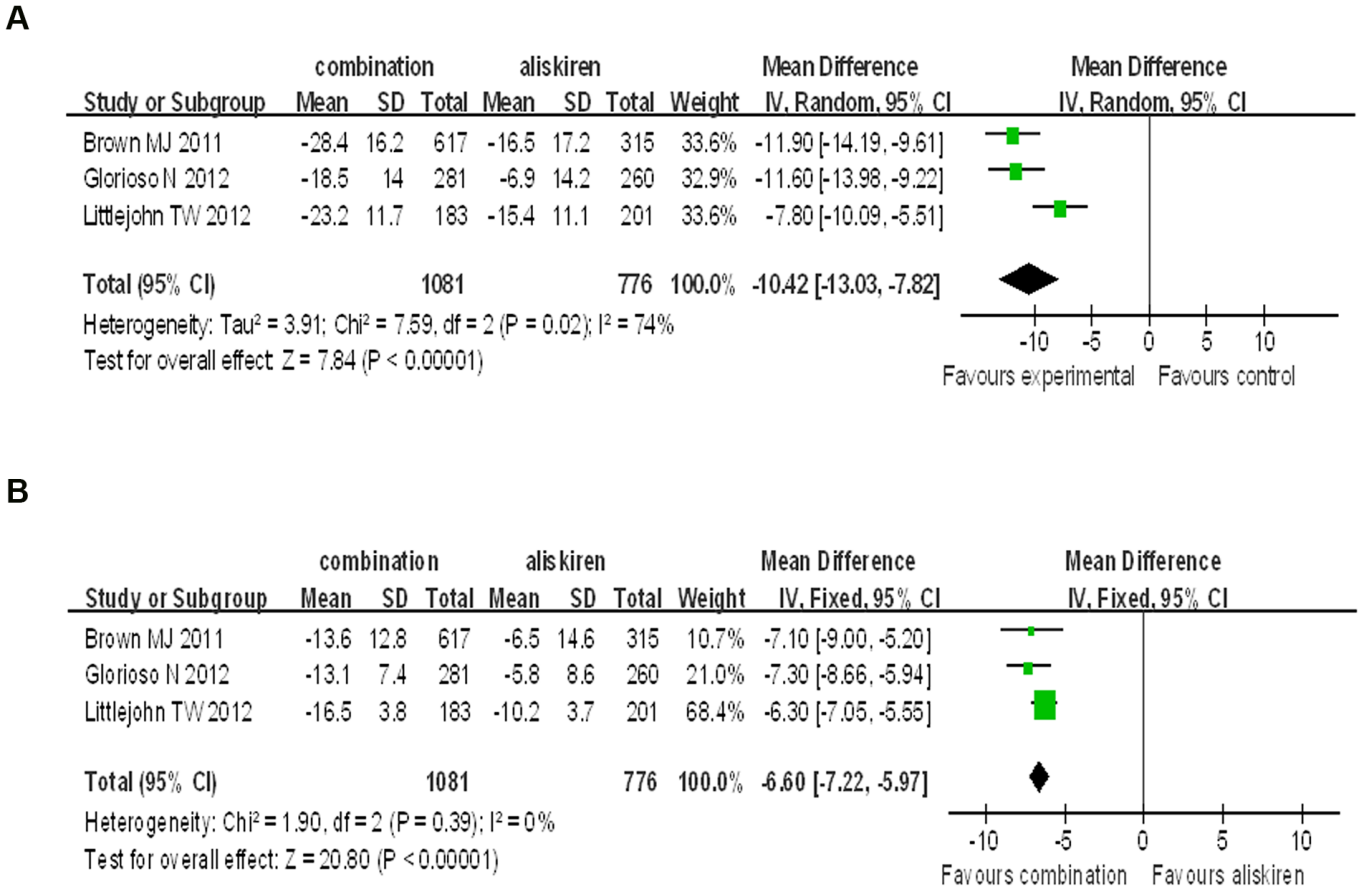
Forest plot of BP reduction efficacy of aliskiren/amlodipine 300/10 mg/d and 300 mg/d aliskiren. (a) Comparison of change in systolic blood pressure (SBP), diastolic blood pressure (DBP) (b).

In all the 3 trials we analyzed, the mean baseline BP for the combination group was 158.35 mmHg for SBP and 94.60 mmHg for DBP; while the mean baseline BP of the aliskiren group was 157.49 mmHg for SBP and 95.59 mmHg for DBP. The final achieved BP of the combination group was 133.55 mmHg for SBP and 80.63 mmHg for DBP; while the final achieved BP of the aliskiren group was 144.68 mmHg for SBP and 88.36 mmHg for DBP. Our analysis showed that aliskiren/amlodipine combination therapy was significantly superior to aliskiren monotherapy on SBP and DBP reduction. (SBP: WMD = −10.42, 95% CI −13.03∼−7.82, P<0.00001; DBP: WMD = −6.60, 95% CI −7.22∼−5.97, P<0.00001). Although no significant heterogeneity was found in the analysis for DBP reduction (P = 0.39, I^2^ = 0%), there was significant heterogeneity in the analysis for SBP (P = 0.02, I^2^ = 74%) (**Figure 2**).

#### 3.2. Combination therapy of aliskiren combined with amlodipine versus amlodipine

**Figure 3:**
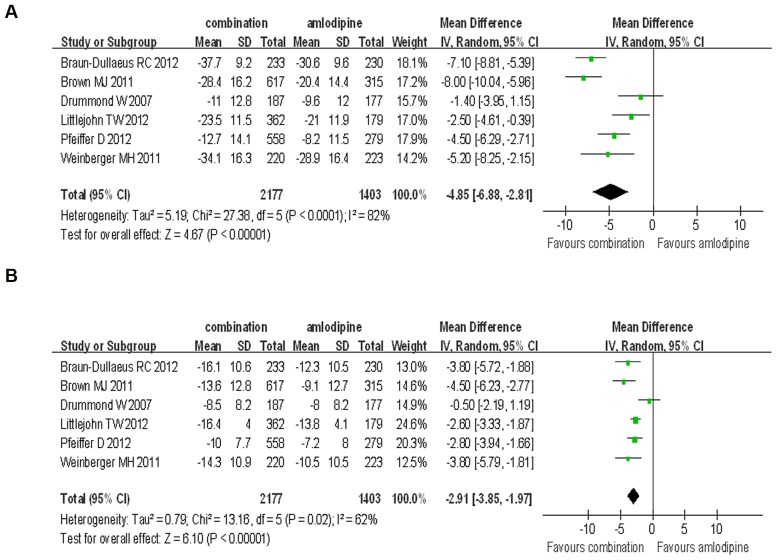
Forest plot of BP reduction efficacy of aliskiren combined with amlodipine and amlodipine monotherapy at any doses. (a) Comparison of change in systolic blood pressure (SBP); (b) Comparison of change in diastolic blood pressure (DBP).

In the comparison of the SBP and DBP reduction efficacy of aliskiren/amlodipine combination therapy versus the amlodipine monotherapy at any dose, the mean baseline BP of combination group was 158.73 mmHg for SBP and 95.00 mmHg for DBP; while the mean baseline BP of amlodipine group was 159.82 mmHg for SBP and 95.76 mmHg for DBP. The final achieved BP of the combination group was 135.15 mmHg for SBP and 82.26 mmHg for DBP; while the final achieved BP of the amlodipine group was 140.21 mmHg for SBP and 85.89 mmHg for DBP. Our analysis showed that the aliskiren/amlodipine combination therapy was significantly superior to amlodipine monotherapy on SBP and DBP reduction (SBP: WMD = −4.85, 95% CI −6.88∼−2.81, P<0.00001; DBP: WMD = −2.91, 95% CI −3.85∼−1.97, P<0.00001), and there was significant heterogeneity in the analysis for SBP and DBP reduction (P<0.0001, I^2^ = 82%; P = 0.02, I^2^ = 62%, respectively) (**Figure 3**).

**Figure 4:**
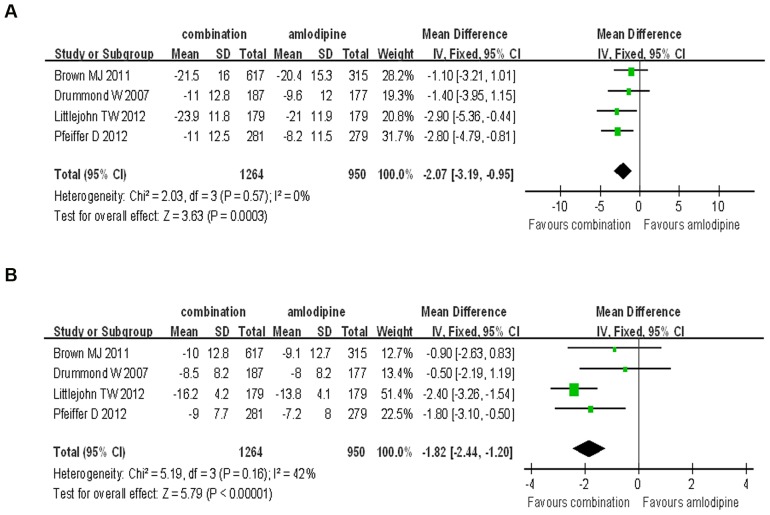
Forest plot of BP reduction efficacy of 150 mg/d aliskiren combined with amlodipine and 10 mg/d amlodipine monotherapy. (a) Comparison of change in systolic blood pressure (SBP); (b) Comparison of change in diastolic blood pressure (DBP).

In the published papers, the dosage of aliskiren varied from 150 mg to 300 mg per day. At first, we compared the BP reduction efficacy of 150 mg/d aliskiren combined with amlodipine and the 10 mg/d amlodipine monotherapy. Aliskiren (150 mg/d) combined with amlodipine was superior to the amlodipine monotherapy in SBP and DBP reduction (SBP: WMD = −2.07, 95% CI −3.19∼−0.95, P = 0.0003; DBP: WMD = −1.82, 95% CI −2.44∼−1.20, P<0.00008). No significant heterogeneity were found in the analysis for SBP and DBP reduction (P = 0.57, I^2^ = 0%; P = 0.16, I^2^ = 42%, respectively) (**Figure 4**).

**Figure 5:**
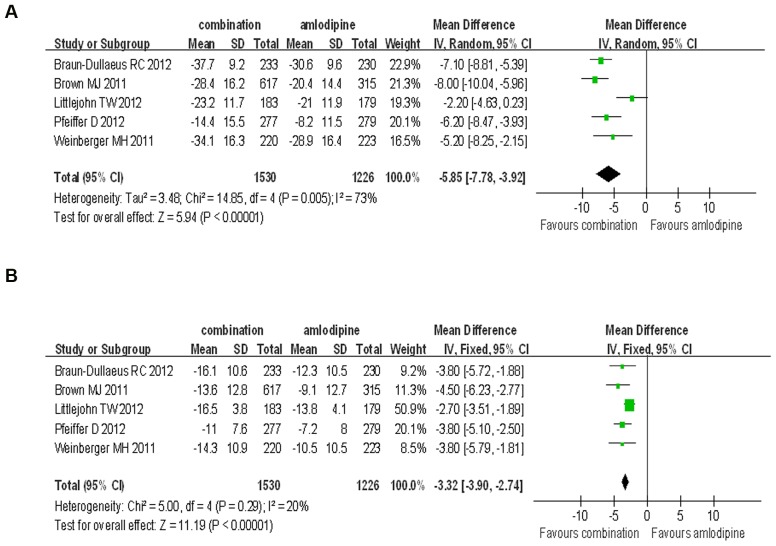
Forest plot of BP reduction efficacy of 300 mg/d aliskiren combined with amlodipine and 10 mg/d amlodipine. (a) Comparison of change in systolic blood pressure (SBP). (b) Comparison of change in diastolic blood pressure (DBP).

Besides, 300 mg/d aliskiren was used in some clinical trials, with four randomized controlled trials included to compare the BP reduction efficacy and safety of the participants using 300 mg/d aliskiren combined with amlodipine or 10 mg/d amlodipine monotherapy. The results showed that the combination therapy was superior to amlodipine monotherapy alone on the reduction of SBP and DBP. (SBP: WMD = −5.85, 95% CI −7.78∼−3.92, P<0.00001; DBP: WMD = −3.32, 95% CI −3.90∼−2.74, P<0.00001) There was significant heterogeneity in the analysis for SBP reduction, but no significant heterogeneity in the analysis for DBP reduction (P = 0.005, I^2^ = 73%; P = 0.29, I^2^ = 20%, respectively) (**Figure 5**).

**Figure 6:**
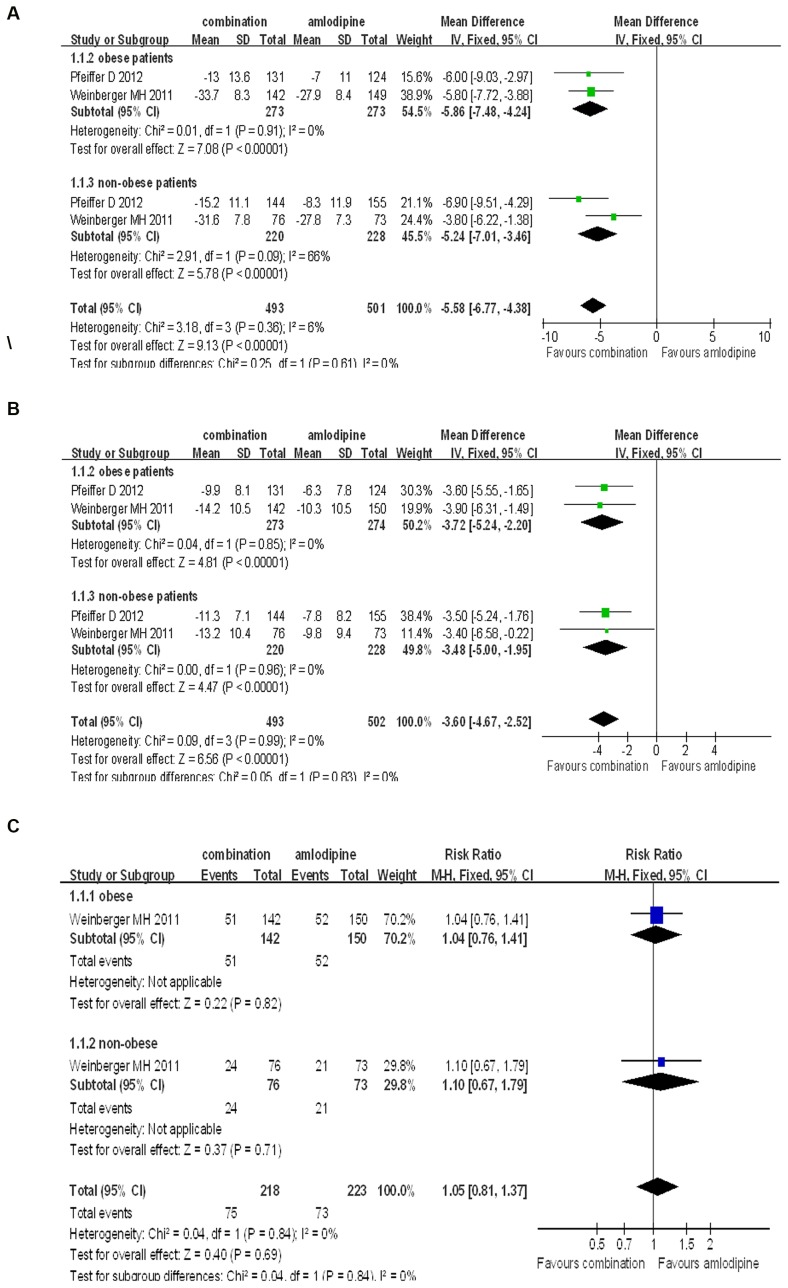
Forest plot of BP reduction efficacy and safety of aliskiren/amlodipine and amlodipine in obese patients and non-obese patients. (a) Comparison of change in systolic blood pressure (SBP). (b) Comparison of change in diastolic blood pressure (DBP). (c) Comparison of adverse events incidence.

In further analysis, we compared combination therapy of aliskiren and amlodipine to monotherapy of amlodipine in either obese patients or non-obese patients. The results showed that the combination therapy was very much superior to amlodipine monotherapy on the reduction of SBP and DBP in obese patients (SBP: WMD = −5.86, 95% CI −7.48∼−4.24, P<0.00001; DBP: WMD = −3.27, 95% CI −5.24∼−2.20, P<0.00001). There was no significant heterogeneity in the analysis for SBP and DBP (P = 0.91, I^2^ = 0%; P = 0.85, I^2^ = 0%, respectively). The rate of adverse effects was found no significant difference between combination group and amlodipine group (RR = 1.04, 0.76∼1.41, P = 0.82). Similar results were found in the subgroup analysis in non-obese-hypertensive patients (SBP: WMD = −5.24, 95% CI −7.01∼−3.46, P<0.00001; DBP: WMD = −3.48, 95% CI −5.00∼−1.95, P<0.00001. Adverse effects: RR = 1.10, 0.67∼1.79, P = 0.71) (**Figure 6**).

**Figure 7:**
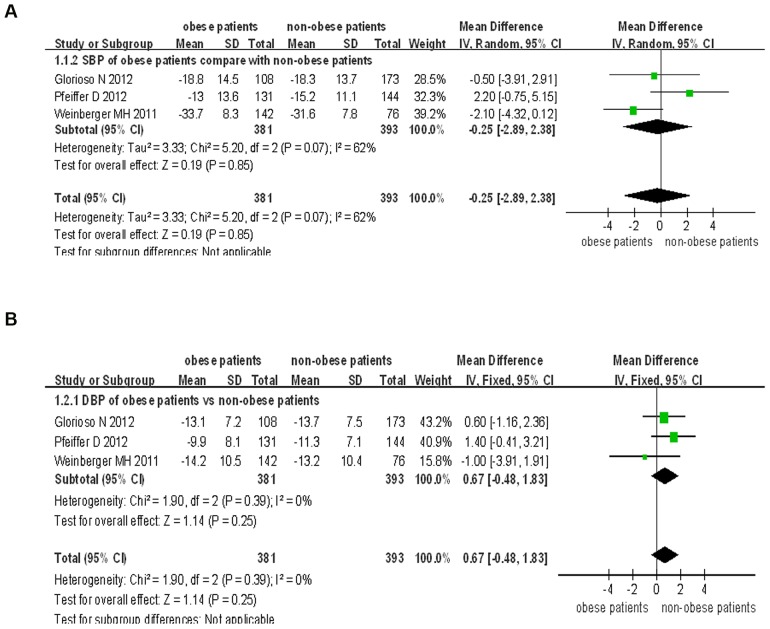
Forest plot of BP reduction efficacy of aliskiren/amlodipine combination therapy in obese patients and that in non-obese patients. (a) Comparison of change in systolic blood pressure (SBP). (b) Comparison of change in diastolic blood pressure (DBP).

We also compared the combination therapy of aliskiren and amlodipine in obese patients to that in non-obese patients. The results showed that there was no difference with regards to the lowering of the blood pressure by the combination therapy in both obese and non-obese subgroups (SBP: WMD = −0.25, 95% CI −2.89∼2.38, P = 0.85; DBP: WMD = 0.67, 95% CI −0.48∼1.83, P = 0.25). There was significant heterogeneity in the analysis for SBP (P = 0.40, I^2^ = 62%), but no significant heterogeneity for DBP (P = 0.39, I^2^ = 0%) (**Figure 7**).

### 4. Safety of Aliskiren Combined with Amlodipine versus Alsikrein or Amlodipine Monotherapy

#### 4.1 Combination therapy of aliskiren with amlodipine versus aliskiren

When comparing the AEs in the aliskiren/amlodipine group with those in the amlodipine monotherapy group, we found no difference for the incidence of AEs, discontinuation due to AEs and serious adverse events between the combination therapy and aliskiren monotherapy (RR = 1.11, 95% Cl 0.87∼1.26, P = 0.53; RR = 1.09, 95% Cl 0.79∼1.49, P = 0.62; RR = 0.95, 95% Cl 0.45∼2.04, P = 0.90, respectively). Only the rate of peripheral edema was higher in the combination group than in the aliskiren monotherapy group (RR = 1.60, 1.22∼2.12, P = 0.0008) (**Table 2**).

**Table 2:** Aliskiren/amlodipine combination therapy versus aliskiren monotherapy on adverse events.

Category	RR	95% CI	p-value
Any AE	1.11	0.80–1.54	0.53
Dicontinuation due to AE	1.09	0.79–1.49	0.62
Any SAE	0.95	0.45–2.04	0.90
Peripheral edema	1.60	1.22–2.12	0.0008
Nasopharyngitis	1.86	0.47–7.35	0.38
Dizziness	1.39	0.71–2.74	0.34
Headache	0.37	0.12–1.17	0.09
Hyperkalaemia	0.64	0.26–1.54	0.32
Hypokalaemia	1.18	0.39–3.55	0.77

Abbreviations: RR, relative risk; CI, confidence interval; AEs, adverse events; SAEs, serious adverse events.

#### 4.2 Combination therapy of aliskiren combined with amlodipine versus amlodipine alone

We next compared the differences in the occurrence of AEs in the aliskiren/amlodipine group with the amlodipine monotherapy group. There were no differences with the rates of AEs or SAEs between the two groups (RR = 1.02, 0.95∼1.10, P = 0.56; RR = 0.71, 0.34∼1.47, P = 0.36, respectively). However, it was interesting to find, that the rate of medication discontinuation due to AEs was lower in the aliskiren/amlodipine group than in the amlodipine monotherapy group (RR = 0.64, 0.50∼0.83, P = 0.0008); moreover, the rates of peripheral edema and hypokalaemia were significantly lower in the combination therapy than that in the amlodipine monotherapy group (RR = 0.78, 0.66∼0.92, P = 0.004; RR = 0.51, 0.27∼0.97, P = 0.04, respectively) (**Table 3**).

**Table 3:** Aliskiren/amlodipine combination therapy versus amlodipine monotherapy on adverse events.

Category	RR	95% CI	p-value
Any AEs	1.02	0.95–1.10	0.56
Dicontinuation due to AEs	0.64	0.50–0.83	0.0008
Any SAEs	0.71	0.34–1.47	0.36
Peripheral edema	0.78	0.66–0.92	0.004
Nasopharyngitis	1.09	0.67–1.78	0.73
Dizziness	1.17	0.75–1.84	0.48
Headache	0.76	0.48–1.22	0.25
Hyperkalaemia	0.77	0.40–1.47	0.42
Hypokalaemia	0.51	0.27–0.97	0.04

Abbreviations: RR, relative risk; CI, confidence interval; AEs, adverse events; SAEs, serious adverse events.

## Discussion

Current guidelines recommend that combination therapy should only be considered in the onset of grade II or a more severe form of hypertension. The combination of CCBs and RAAS inhibitors, including angiotensin converting enzyme inhibitor (ACEI) and AT_1_ receptor blockers (ARBs), is a common anti-hypertension therapy. However, recent studies also report the disadvantages of the use of ACEIs or ARBs as compared with the use of aliskiren. It has been reported that the long term treatment with ACEIs can cause the recovery of the plasma level of angiotensin II which may be induced by the compensatory increase in plasma renin activity (PRA) and angiotensin I [20], [21]. This recovery could attenuate the effects of ACEIs on RAAS blockade. Similar phenomena occurs with ARBs, the use of ARBs can cause the increase of angiotensin II and may over-stimulate the unblocked AT_1_ receptors. As an inhibitor of renin, aliskiren inbitits the down-stream signals, including PRA, angiotensin I and angiotensin II, which may in turn exert some benefits in lowering hypertension. Both in combination therapy [9] and in monotherapy [22], aliskiren-based therapy has been proved to produce a greater BP reductions with less AEs compared with other RAAS inhibitors. The aliskiren-based combination therapies including aliskiren combined with CCBs have been getting more and more attention in recent years.

Does the combination therapy of aliskiren and amlodipine provide a more efficient control of BP than monotherapy? Whether the new therapy strategy leads to more AEs or not is not yet known. Many clinical trials have studied the issue, with varying results being demonstrated. Most trials concluded that the BP reduction using combination therapy was significantly greater than that seen in monotherapy, while Drummond and colleagues found the effect similar to that of amlodipine monotherapy [13]. Our meta-analysis supported the conclusion that the efficiency of combination therapy is much superior to that of monotherapy. We think the reason why the Drummond research got a different conclusion may be because the study compared a lower dosage of the combination therapy with the amlodipine monotherapy, and with the shortest follow-up time (6 weeks in Drummond’s research, compared to 8 weeks in the other researches in the low dosage group).

Many preclinical researches on the different medicines also indicated that combination therapy might be superior to monotherapy. The RAAS inhibitors and CCBs exert BP lowering effects via independent mechanisms. CCBs have a direct effect on vascular smooth muscle relaxation mainly by the effect of their inhibitory action on voltage-gated calcium channels; while RAAS inhibitors directly blockade the RAAS whose excessive activity could cause primary hypertension. The combination therapy using RAAS inhibitors with CCBs can control BP via different targets. Thus, combination therapy may provide a more effective method in controlling BP than monotherapy. In addition, CCBs also has effects in increasing global sympathetic activity and decreasing parasympathetic activity [23], whereas many RAAS inhibitors can increase parasympathetic activity [24], [25], suggesting that the effects of CCBs on the autonomic nervous system may be mitigated when combined with RAAS inhibitors. Amlodipine, a kind of CCB, has vasodilatory effect partly through the stimulation of nitric oxide release from blood vessels, which is independent of its effect on calcium channels, might by activation of B2 receptor. Moreover, amlodipine may be related to an increase in local bradykinin through the inhibition of ACE [26], which may enhance the effect of RAAS inhibitors. Furthermore, amlodipine and aliskiren may also exert beneficial effects on the endothelium, such as improved nitric oxide availability. It has been suggested that these agents may have complementary effects on the vascular wall [27]–[29]. Since CCBs and RAAS inhibitors have these complementary effects on the renin system, sympathetic activity and vascular endothelial function, they may not only be more effective in BP reduction, but also eliminate some of the adverse effects.

The most common of the adverse effects includes peripheral edema, headache, dizziness and nasopharyngitis, we found no significant differences between aliskiren/amlodipine combination therapy and either of the monotherapies with regards to total adverse effects. However, the type of adverse effect most likely to occur might change in the different group. In the group of aliskiren/amlodipine versus aliskiren, the rate of peripheral edema in the combination group is higher than that in the aliskiren group, but the rate of peripheral edema is significantly lower in the combination group than that in the amlodipine group. These results indicate that peripheral edema is most likely caused by amlodipine, and could be attenuated by aliskiren. It is also interesting to find that in the comparison of aliskiren/amlodipine combination therapy with amlodipine monotherapy, the rate of hypokalaemia is significantly lower in the combination therapy than that in the amlodipine monotherapy. This result may be caused by the potassium sparing effect of aliskiren [30], while the amlodipine monotherapy might stimulate potassium excretion. There are also some limitations in the analysis of adverse events. In the comparison between the aliskiren/amlodipine combination therapy and the aliskiren monotherapy, the trials we pooled to analyze the AEs wee few, and the adverse effects of nasopharyngitis, dizziness and headache are only reported in the Weinberger MH’s trial. It might need more RCTs, which compare the AEs in the aliskiren/amlodipine combination therapy, to make the results on this issue more convincing. Furthermore, the included trials only evaluate the short-term effects of those therapies, whether or not the long-term effects have the similar phenomenon need further studies to be included in the future.

Previous studies have reported that the anti-hypertensive medicines might have different effects on the obese and non-obese patients. For example, Kato J et al found that the combination of ARB and CCB had a greater BP lowering effect in non-obese than in obese patients [31]. To investigate further whether or not a similar phenomenon occurs in the obese and non-obese patients, we did a meta-analysis study of the subgroup. When it comes to the comparison of combination therapy efficacy in patients who are obese or non-obese, the trials showed different conclusions: Glorioso N and Pfeiffer D concluded that the BP lowering efficiency of the combination therapy in obese-patients was comparable with that in non-obese patients [16], [18], while Weinberger MH showed that the efficiency was slightly superior in obese patients than in non-obese patients [14]. Our meta-analysis showed that there is no significant difference in the efficacy of combination therapy between obese patients and non-obese patients. The reason why Weinberger MH obtained a different conclusion may be due to the sample size of two groups not being the same. In the trial of Weinberger MH, the obese group had 142 participants while the non-obese group had only 76 participants which might not be sufficient to represent the characteristics of non-obese patients. This may have resulted in lack of statistical power to detect some between-treatment differences within these subgroups that may have precipitated the difference in the conclusions. In the same token, the number of clinical trials which were included in our meta-analysis is still relatively small, so we may still need more trials to get a more convincing and clearer conclusion.

In conclusion, aliskiren/amlodipine combination therapy provides a more effective blood pressure reduction without increase in the occurrence of adverse events as compared with monotherapy of those medicines. No difference is found between obese patients and non-obese patients undergoing the combination therapy. Due to lack of a long-term follow-up and cardiovascular event records, further experiments need to be done in the future.

## Supporting Information

Figure S1 **Funnel plot of the studies.** (a) Effects of aliskiren/amlodipine combination therapy vs that of aliskiren monotherapy. (b) Adverse events of aliskiren/amlodipine combination therapy vs that of amlodipine monotherapy at any doses. (c) Combination therapy of 150 mg/d aliskiren and amlodipine vs 10 mg/d amlodipine monotherapy. (d) Combination therapy of 300 mg/d aliskiren and amlodipine vs 10 mg/d amlodipine monotherapy. (e) Combination therapy of aliskiren and amlodipine vs monotherapy of amlodipine in either obese patients or non-obese patients. (f) Combination therapy of aliskiren and amlodipine in obese patients vs that in non-obese patients. (g) Adverse events of aliskiren/amlodipine combination therapy vs that of aliskiren monotherapy. (h) Adverse events of aliskiren/amlodipine combination therapy vs that of amlodipine monotherapy at any doses. MD: mean difference.
(TIF)

Table S1 **Risk of bias summary of included studies.**
(DOC)

Checklist S1 **PRISMA checklist.**
(DOC)
