# Supplementary figures and images for: Aliskiren and Amlodipine in the Management of Essential Hypertension: Meta-Analysis of Randomized Controlled Trials
Source: PLoS One. 2013 Jul 29;8(7):e70111. doi: 10.1371/journal.pone.0070111 (PMC3726495; doi:10.1371/journal.pone.0070111)

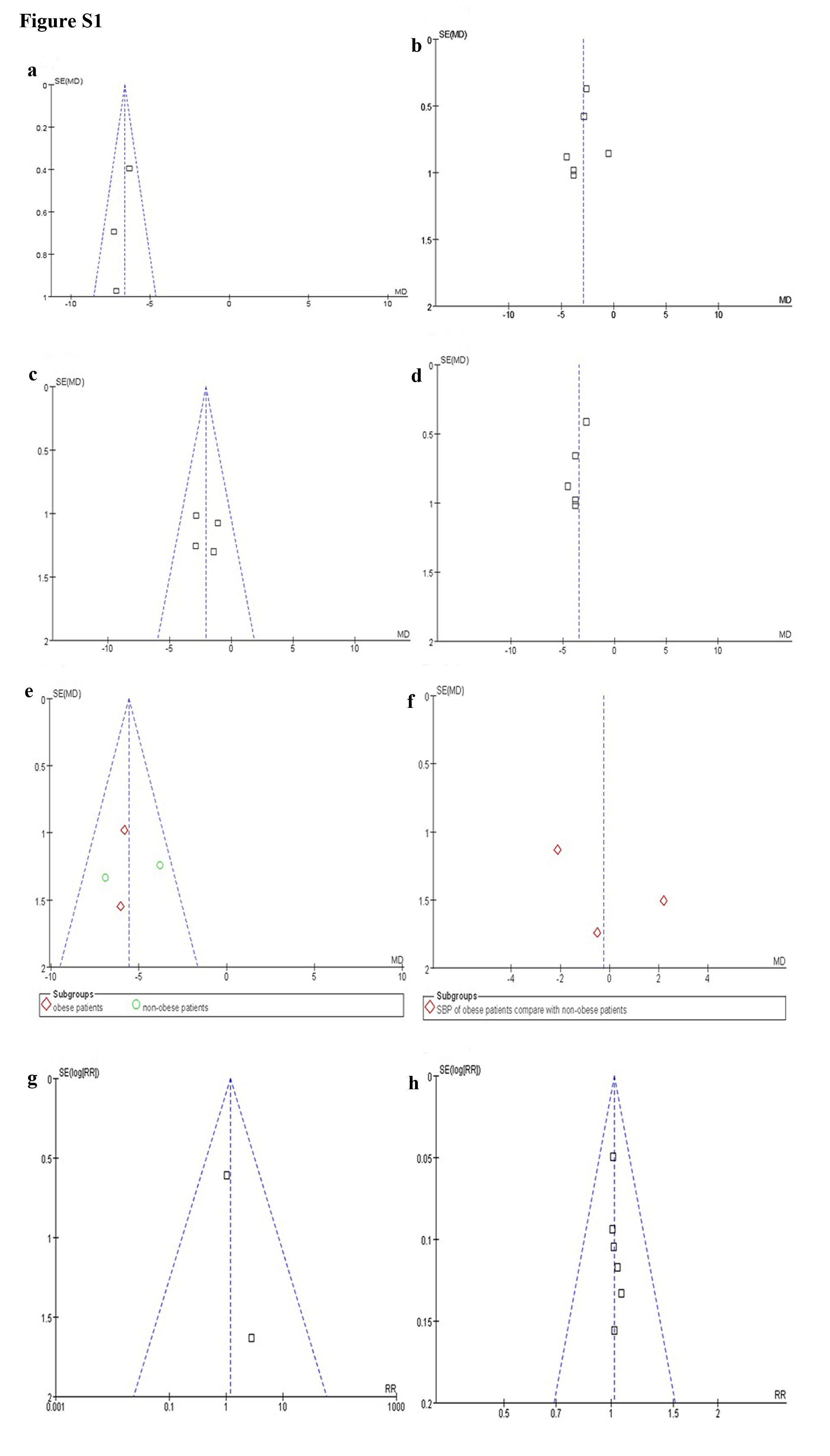

Supplement: Figure S1 — Funnel plot of the studies. (a) Effects of aliskiren/amlodipine combination therapy vs that of aliskiren monotherapy. (b) Adverse events of aliskiren/amlodipine combination therapy vs that of amlodipine monotherapy at any doses. (c) Combination therapy of 150 mg/d aliskiren and amlodipine vs 10 mg/d amlodipine monotherapy. (d) Combination therapy of 300 mg/d aliskiren and amlodipine vs 10 mg/d amlodipine monotherapy. (e) Combination therapy of aliskiren and amlodipine vs monotherapy of amlodipine in either obese patients or non-obese patients. (f) Combination therapy of aliskiren and amlodipine in obese patients vs that in non-obese patients. (g) Adverse events of aliskiren/amlodipine combination therapy vs that of aliskiren monotherapy. (h) Adverse events of aliskiren/amlodipine combination therapy vs that of amlodipine monotherapy at any doses. MD: mean difference. (TIF) [file pone.0070111.s001.tif]
