# Supplementary material for: Aliskiren and Amlodipine in the Management of Essential Hypertension: Meta-Analysis of Randomized Controlled Trials
Source: PLoS One. 2013 Jul 29;8(7):e70111. doi: 10.1371/journal.pone.0070111 (PMC3726495; doi:10.1371/journal.pone.0070111)
Supplement: Table S1 — Risk of bias summary of included studies. (DOC) [file pone.0070111.s002.doc]

**Supplementary Tables**

**Table S1 Risk of bias summary of included studies**.

| Study | Design | Adequate sequence generation | Allocation concealment | Blinding of research personnel | Blinding of outcome | Incomplete outcome data addressed | Free of selective reporting | Free of other bias | Risk of bias |
| --- | --- | --- | --- | --- | --- | --- | --- | --- | --- |
| Brown MJ | RCT | Low risk | Low risk | Low risk | Low risk | Low risk | Low risk | Low risk | Low risk |
| Weinberger MH | RCT | Moderate risk | Moderate risk | Low risk | Low risk | Low risk | Low risk | Low risk | Moderate risk |
| Pfeiffer D | RCT | Low risk | Low risk | Low risk | Low risk | Low risk | Low risk | Low risk | Low risk |
| Drummond W | RCT | Moderate risk | Moderate risk | Moderate risk | Moderate risk | Low risk | Low risk | Low risk | Moderate risk |
| Glorioso N | RCT | Low risk | Low risk | Low risk | Low risk | Low risk | Low risk | Low risk | Low risk |
| Braun-Dullaeus RC | RCT | Moderate risk | Moderate risk | Low risk | Low risk | Low risk | Low risk | Low risk | Moderate risk |
| LittlejohnTW | RCT | Low risk | Low risk | Low risk | Low risk | Low risk | Low risk | Low risk | Low risk |
